# Playing HAVOK on the Chaos Caused by Internet Trolls

**DOI:** 10.21203/rs.3.rs-2843058/v1

**Published:** 2023-04-25

**Authors:** Elena Martynova, Hudson Golino, Steven Boker

**Affiliations:** University of Virginia

**Keywords:** nonlinear dynamical systems, time series analysis, text mining, HAVOK, dynamic EGA, Russian trolls, Twitter trolls, 2016 presidential election

## Abstract

Trump supporting Twitter posting activity from right-wing Russian trolls active during the 2016 United States presidential election was analyzed at multiple timescales using a recently developed procedure for separating linear and nonlinear components of time series. Trump supporting topics were extracted with DynEGA (Dynamic Exploratory Graph Analysis) and analyzed with Hankel Alternative View of Koopman (HAVOK) procedure. HAVOK is an exploratory and predictive technique that extracts a linear model for the time series and a corresponding nonlinear time series that is used as a forcing term for the linear model. Together, this forced linear model can produce surprisingly accurate reconstructions of nonlinear and chaotic dynamics. Using the R package havok, Russian troll data yielded well-fitting models at several timescales, not producing well-fitting models at others, suggesting that only a few timescales were important for representing the dynamics of the troll factory. We identified system features that were timescale-universal versus timescale-specific. Timescale-universal features included cycles inherent to troll factory governance, which identified their work-day and work-week organization, later confirmed from published insider interviews. Cycles were captured by eigen-vector basis components resembling Fourier modes, rather than Legendre polynomials typical for HAVOK. This may be interpreted as the troll factory having intrinsic dynamics that are highly coupled to nearly stationary cycles. Forcing terms were timescale-specific. They represented external events that precipitated major changes in the time series and aligned with major events during the political campaign. HAVOK models specified interactions between the discovered components allowing to reverse-engineer the operation of Russian troll factory. Steps and decision points in the HAVOK analysis are presented and the results are described in detail.

## Introduction

Strategic misinformation has been spiraling out of control with intranational and international actors attempting to manipulate social media users for political and commercial reasons. Fortunately, social media platforms keep records that can be aggregated into publicly available datasets and subjected to quantitative analysis. Modern quantitative techniques can be a key to extracting, understanding, and predicting misinformation for regulation and prevention.

In the United States, online activities were used to influence public opinion and voters during the 2016 presidential campaign, with attacks occurring before and during the electoral process. Twitter accounts linked to the Russian state-sponsored Internet Research Agency (IRA) were used to try to divide voters on a wide range of issues and data from these accounts were released by the United States Congress after an investigation (Zannettou, Caulfield, De Cristofaro, et al., 2019). A large database containing almost 3 million Twitter posts from the years 2013 to 2018 and originating from 2843 unique IRA-linked twitter accounts is available online (Roeder, 2018).

Recent literature on Twitter disinformation is vast, but still insufficient. Different aspects of online social media disinformation have been actively researched over the recent years through qualitative and quantitative methods with the purpose of the detection of malevolent accounts and their discernment from one another. The most studied troll characteristics include linguistic profiles (e.g., linguistic complexity and emotional charge of the language; Addawood, Badawy, Lerman, & Ferrara, 2019; Monakhov, 2020; Lundberg & Laitinen, 2020; Uyheng & Carley, 2021b; Golino, Christensen, Moulder, Kim, & Boker, 2022; Uyheng, Moffitt, & Carley, 2022; Zannettou, Caulfield, Setzer, et al., 2019), automation levels (human trolls, bot trolls, or human-bot trolls; Achimescu & Sultanescu, 2020; Alsmadi & O’Brien, 2020; Badawy, Ferrara, & Lerman, 2018; Bastos & Mercea, 2018; Broniatowski et al., 2018; Chu, Gianvecchio, Wang, & Jajodia, 2010, 2012; Chu, Gianvecchio, & Wang, 2018; Paavola, Helo, Jalonen, Sartonen, & Huhtinen, 2016; Uyheng et al., 2022), sponsorship origins (e.g., Russian, Chinese, Iranian state-sponsored, or private entity sponsored; Badawy et al., 2018; Im et al., 2020; Ong & Cabaijes, 2019; Tan, 2020; Tapsell, 2021; Uyheng et al., 2022; Zannettou, Caulfield, De Cristofaro, et al., 2019; Zannettou, Caulfield, Setzer, et al., 2019), and potential taxonomies (e.g., provocators, fearmongers, and hashtag gamers; Berghel & Berleant, 2018; Gorwa & Guilbeault, 2020; Linvill & Warren, 2020b; Luceri, Deb, Badawy, & Ferrara, 2019; Orabi, Mouheb, Al Aghbari, & Kamel, 2020; Shao et al., 2018; Stella, Ferrara, & De Domenico, 2018). Studies of these characteristics provide valuable insight into the general functioning of social media trolls.

For instance, troll messages have been found to possess lower cognitive complexity, more abusive language, and greater targeting of named entities and identities than messages from real social profiles (Uyheng et al., 2022). Trolls have been found to be used alongside bots by state actors, with bots being used for automated amplification of the troll messages (Alsmadi & O’Brien, 2020; Badawy et al., 2018; Bastos & Mercea, 2018; Broniatowski et al., 2018). When used separately trolls have been found to have distinct characteristics from bots, with bots disproportionately dominating right-leaning media outlets and trolls dominating media outlets with bipartisan trust (Uyheng et al., 2022). International state-sponsored troll accounts have been found to be dominated by Chinese (Uyheng et al., 2022). Different state-sponsored accounts were found to employ similar operation tactics, but utilize distinctive language (Badawy et al., 2018; Lundberg & Laitinen, 2020). Private-entity-sponsored trolling accounts tend to employ tactics that overlap with the state-sponsored accounts’ and are often underrepresented in the online trolling literature, since they are often not categorized as trolls. These accounts are referred to by a range of different names such as “buzzers” and “sybils” (Keller, Schoch, Stier, & Yang, 2017; Ong & Cabaijes, 2019; Orabi et al., 2020; Tan, 2020; Tapsell, 2021; Udupa, 2018; Uyheng, Magelinski, Villa-Cox, Sowa, & Carley, 2020; Uyheng et al., 2022). Not to be confused with a range of troll-referring names that represent their taxonomies. These taxonomies categorize troll accounts that tend to have specialized tasks (e.g., to divide discussants, to attack undesired opinions, or to dissipate false narratives; Berghel & Berleant, 2018; Linvill & Warren, 2020b). Such taxonomies, when identified correctly, greatly aid in troll account detection, since real accounts rarely possess such uniform behaviors (Linvill & Warren, 2020b).

Although recent findings contribute to advancing the understanding of online manipulations by social media trolls, they can have conflicting definitions of core concepts and yield contradictory findings. Some findings are highly context dependent and might be counterproductive when generalized across different contexts (Gorwa & Guilbeault, 2020; Uyheng et al., 2022), and some findings are only relevant for fixed periods of time, since troll strategies are evolving (Alizadeh, Shapiro, Buntain, & Tucker, 2020; Alsmadi & O’Brien, 2020; Cresci, 2020; Zannettou, Caulfield, Setzer, et al., 2019). For instance, in some studies trolling has been defined by the consistently disruptive, provocative, and malevolent nature of online interactions (Badawy et al., 2018; Cheng, Bernstein, Danescu-Niculescu-Mizil, & Leskovec, 2017; Monakhov, 2020; Zannettou, Caulfield, Setzer, et al., 2019), while other studies contain troll definitions that rely solely on the nature of their sponsorship, i.e. state-sponsored accounts (Im et al., 2020; Zannettou, Caulfield, De Cristofaro, et al., 2019; Zannettou, Caulfield, Setzer, et al., 2019) or private-institution-sponsored accounts (Ong, 2020; Ong & Cabaijes, 2019), regardless of their interaction characteristics.

Even when researchers agree on defining trolls by the malevolent nature of their interactions, belonging to the troll class is often determined by the thin lines between the subtypes of expression of malevolence with hate speech (Davidson, Warmsley, Macy, & Weber, 2017; Kocoń et al., 2021; Uyheng & Carley, 2020, 2021b) and cyber-agression (Rosa et al., 2019) classified as phenomena different from trolling (Uyheng et al., 2022). Moreover, there is no clear agreement on the role of automation in the definition of trolls, with some researchers classifying trolls as exclusively human actors (Bastos & Mercea, 2018; Broniatowski et al., 2018), whereas others allowing bots into the definition (Paavola et al., 2016). While such inconsistencies exist, research findings are likely to differ due to the study of potentially different phenomena, especially when they are immersed into different contexts (Gorwa & Guilbeault, 2020; Orabi et al., 2020). For instance, bots that might or might not be classified as trolls have been found to be associated with use of abusive language in some contexts (Stella et al., 2018; Uyheng & Carley, 2021a; Uyheng et al., 2022), and having no correlation with abusive language (Uyheng et al., 2020) in other contexts. Even contexts that are similar at the present time will inevitably change with time, in turn also altering the interests of the state and private entity sponsors, who will modify their approaches accordingly (Zannettou, Caulfield, Setzer, et al., 2019; Alizadeh et al., 2020; Alsmadi & O’Brien, 2020; Cresci, 2020; Llewellyn, Cram, Hill, & Favero, 2019). The definitions and thresholds, even if clearly established, will need to be reconsidered and adjusted accordingly, making it necessary to continue the research of trolling with respect to time.

Although some troll and social bot studies consider select temporal aspects in their analyses (e.g., Llewellyn et al., 2019; Park, Strover, Choi, & Schnell, 2021; Rajtmajer, Simhachalam, Zhao, Bickel, & Griffin, 2020; Zannettou et al., 2020; Zannettou, Caulfield, De Cristofaro, et al., 2019) and some temporal aspects of trolling are utilized in machine learning troll detection algorithms (e.g., Chu et al., 2012, 2018; Engelin & De Silva, 2016; Fornacciari, Mordonini, Poggi, Sani, & Tomaiuolo, 2018; Galán-García, Puerta, Gómez, Santos, & Bringas, 2016), studies that examine trolling activities with respect to both short and long timescales are rare. We have not found any studies that research troll activity across multiple timescales. The current article examines temporal aspects of the Russian trolling activities across multiple timescales simultaneously, with timescales ranging from intraday to monthly events. Considering a continuum of timescales and algorithmically determining the timescales that are the most relevant to the internal governance of the troll institutions and events that incite their activity may yield better results than selecting the timescale of focus by guesswork, habit or convenience. Modern quantitative techniques designed for continuous time series analysis, such as HAVOK (Hankel Alternative View of Koopman; Brunton, Brunton, Proctor, Kaiser, & Kutz, 2017) can yield insights into temporally relevant communicative strategies across different timescales when qualitative text data is converted into time series of topical word frequencies.

HAVOK analysis was derived from control theory by an interdisciplinary team of researchers at UW Seattle. HAVOK is a powerful tool for modeling nonlinear and chaotic time series by decomposing them into intermittently forced linear systems. A forcing parameter allows HAVOK to demarcate regions where a time series is approximately linear from those that are nonlinear, with the forcing extrema often preceding shifts in the time series and aligning with the contextual events.

**Figure 1.**
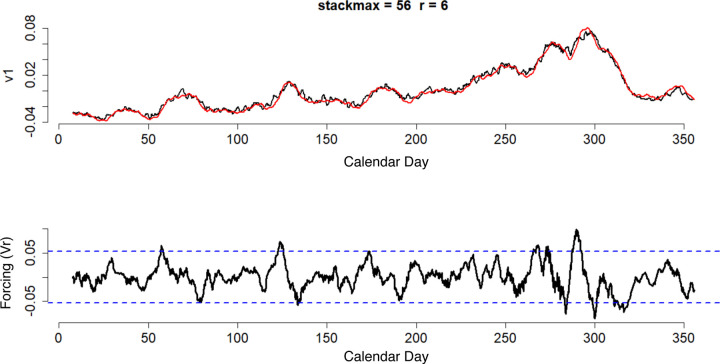
Output from a selected HAVOK model of topical network scores of Trump supporting Russian troll tweets. (a) SVD derived major latent time series of the sum of the mean network scores of the 3 Trump supporting topics in right wing troll posts from IRA in black and HAVOK-predicted latent time series in red. (b) Nonlinear forcing term.

Through a few mathematical steps HAVOK approximates a highly nonlinear system in a state-space representation that consists of a set of components that represent dominant linear dynamics and a forcing term that represents intermittent contribution of nonlinearities. The forcing term (plotted as $V_{r}$ in Figure 1-b) indicates the magnitude of nonlinear behavior exhibited by the system at each timepoint. In Figure 1-a, $v_{1}$ represents major linear component of the measured time series. It is a reconstruction of the major latent component of the measured time series, which approximates the original time series. It is noteworthy that forcing term peaks often tend to slightly precede the occurrence of major events in the measured time series.

It is also possible to generalize HAVOK results beyond training data, predict nonlinear events, and predict a system’s response to nonlinear events. In the context of the Russian Twitter troll analysis, political events that incited the cascades of topical Twitter posts may be identified by alignment with the nonlinear forcing activity. Structural elements of the HAVOK model can help us understand the functioning of the “troll factory,” and those findings can be generalized and used for future predictions.

## Methods

### Data

The data that we used was extracted by Linvill and Warren (2020a) and posted online by the FiveThirtyEight team (Roeder, 2018). The original dataset contains almost 3 million Twitter posts, by 2843 unique accounts, dating from January 2013 to May 2018. The accounts are classified by Linvill and Warren (2020a) into 5 taxonomies: right troll, left troll, news feed, hashtag gamer, and fearmonger. Only right troll and left troll accounts were used in our data preprocessing step, with right trolls representing trolls who tend to promote right-wing values, and left trolls representing trolls who tend to promote left-wing values. Only posts (not including retweets) dating from January 2016 to January 2017 were used. Only accounts that contained at least 50 posts were used, resulting in a total number of posts being 276,752 (78.32% from left trolls). The resulting 236 accounts included in the analysis had a substantial number of followers (median = 877, mean = 4838, 12.37% have more than 10,000 followers), and therefore could be considered influential (Golino et al., 2022; Zannettou, Caulfield, De Cristofaro, et al., 2019).

### Preprocessing

The data were preprocessed as described in Golino et al. (2022). Data from right and left-leaning trolls were pre-processed using the text mining package tm() from R (Theußl, Feinerer, & Hornik, 2012). The sparsity of the corpus of words was decreased using a sparsity threshold of .9935, resulting in 168 unique words. TMFG network and DynEGA were applied using 10 embedded dimensions. Arguments to ‘‘dynEGA’’ were set as follows: time-lag τ=1 and time between successive observations δ=1. The correlation first derivatives were used to estimate the network. The level of analysis was set to *population*, resulting in estimated topics that represent the mean structure of the population.

**Figure 2.**
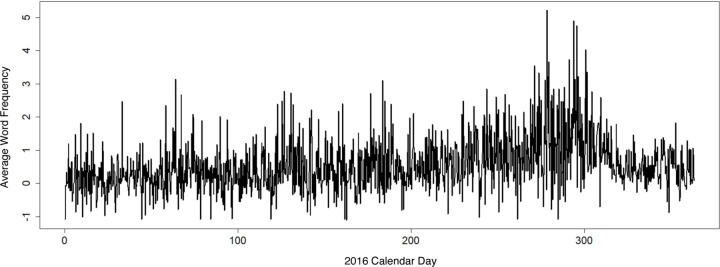
Time series of the sum of the average network scores for the 3 Trump supporting topics (“Money and America/MAGA,” “Supporting Trump for President,” and “Take Back the Country”) in right wing troll posts during the calendar year 2016 within 4 time intervals within each day (00:00–05:59, 06:00–11:59, 12:00–17:59, and 18:00–23:59 Eastern Standard Time).

By estimating the topics DynEGA identified clusters of right-wing and left-wing troll tweet words whose frequencies were changing together in time, identifying dynamic latent topics. HAVOK was applied to the tweet contents that fell into particular latent topics. Topic words whose network scores were used in the analysis belonged to one of the three identified Trump supporting topics: “Money and America/MAGA,” “Supporting Trump for President,” and “Take Back the Country.” Figure 2 plots the time series of the sum of the average network scores for the 3 Trump supporting topics in right-wing troll posts during calendar year 2016 aggregated within 4 within-day time intervals (00:00–05:59, 06:00–11:59, 12:00–17:59, and 18:00–23:59 Eastern Standard Time).

### HAVOK Analysis

HAVOK analysis was performed using the **havok** R package (Moulder & Martynova, 2020). The **havok** package is currently available to download from Github (https://github.com/RobertGM111/havok).

How exactly does HAVOK operate?^1^ In short, dynamics of a system exist in a high dimensional space. We measure the system along one of the dominant axes in time and obtain a measured time series of the system. In psychology, this is what we have and where we startŮwe measure the time series of a phenomenon of interest. In the current case, we use the average network scores of the 3 Trump supporting topics in the right-wing troll tweets.

First we arrange our measured time series into a Hankel matrix, equivalent to the transpose of a time-delay-embedded matrix with lag 1 (Sauer, Yorke, & Casdagli, 1991), where each row contains a segment of the measured time series that is shifted forward by one element from the row above it. Doing this is equivalent to folding up our measured time series and representing it in a higher dimensional space. It is relevant that the time series segments that we are shifting with each row can be of any arbitrary length, also meaning that our Hankel matrix can have an arbitrary number of rows. The number of rows in the Hankel matrix is one of the hyperparameters in HAVOK, and it is directly related to the time-scale on which the model will focus. The higher the number of rows, the lower the frequency of events on which the model will focus, and the smoother the predicted time series will be.

The next step consists of applying singular value decomposition (SVD) to the Hankel matrix H such that,

(1) $$H=U\Sigma V^{\prime }$$

where U is a matrix containing the orthogonal left singular vectors, Σ is a diagonal matrix containing the singular values, and $V^{\prime }$ is the transpose of the matrix containing the right singular vectors. Singular value decomposition is analogous to principal component analysis. If we imagine that wrapping measured time series into a Hankel matrix is analogous to folding and embedding our time series into a higher dimensional space, then application of SVD would be equivalent to decomposing and orthogonalizing those time series in this high dimensional space.

Columns of U contain orthogonal eigen-time series that provide a Koopman-invariant measurement space for the attractor that we are reconstructing in the high dimensional space. When plotted, columns of U may resemble sequential polynomials which extract sequential derivatives when the time series has intrinsic dynamics. If the time series is primarily coupled to stationary cyclic external forcing, such as circadian cycles, then columns of U may appear as sinusoids of increasing wavelength resembling Fourier modes.

The V-transpose matrix contains latent time series components of the measured time series. Each row, $\left(v_{1},v_{2},v_{3},\ldots \right)$, represents a latent time series with the series ordered by their contribution towards the composition of the measured time series in terms of variance. Rows of V-transpose are directly related to the shapes of the columns of U. For instance, if second column of U is of the shape of the quadratic polynomial equivalent for the second derivative, then the latent time series contained in the second row of transpose will approximate the second derivative of the original measured time series.

**Figure 3.**
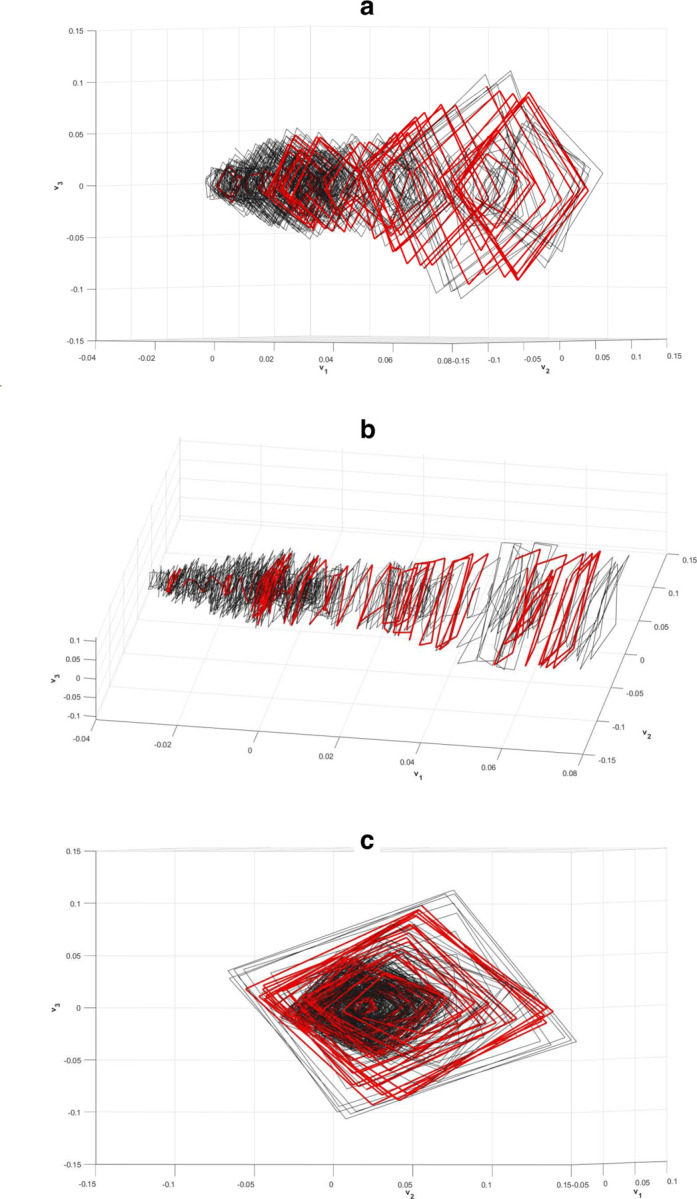
Three views of the three dimensional projection of the HAVOK-reconstructed attractor with nonlinear regions highlighted in red. a) view highlighting the general shape of the attractor. b) view highlighting the separations between nonlinear regions. c) view highlighting quadrangular cycles and the phase shift.

By plotting the first three of these latent time series on mutually orthogonal axes ($v_{1}$, $v_{2}$, & $v_{3}$ in Figure 3) we can reconstruct a three dimensional projection of the attractor that will be *diffeomorphic* to the first three dimensions of the true system attractor. In this context, diffeomorphic means that the true attractor is analogous in its behavior patterns at each corresponding time point to the reconstructed attractor.

The SVD component matrices are then truncated up to the *r* number of components that are needed to accurately approximate the modeled system. Model degree *r* is a hyperparameter in the HAVOK algorithm that helps separate informative model components from noise. So, truncation here also serves as denoising. In theory, the higher the complexity of the system, the higher the model degree will be required. However, real life systems are almost always very complex, and the model degree usually reflects how much of that complexity we can explain with our model, given the quality of the data.

Rows of the truncated V-transpose matrix are used to construct the regression model that will serve as the basis for the HAVOK state-space model. To accomplish this, the first order derivatives of the rows of the truncated V-transpose matrix are regressed onto the rows of the truncated V-transpose matrix. If a system is linear, this regression model will represent it well. But if a system has any nonlinearities, it will not provide a good model fit. To accommodate nonlinear cases, the last column of the regression matrix that contains coefficients for the *r*^*th*^ row of the truncated V-transpose matrix needs to be separated and added to the regression model built from the first r−1 elements as a forcing term, resulting in a HAVOK state-space model like the one shown in Equation 2. The row vector, $v_{r}$, serves as the nonlinear forcing term, indicating the magnitude of nonlinear behavior exhibited by the system at each timepoint. High magnitude forcing bursts tend to precede or coincide with prominent events in the measured time series.

The HAVOK model output now contains predicted linear components of the system, with the major linear component being a reconstruction of the major latent component of the measured time series from the SVD, $v_{1}$ from the V-transpose matrix. This major latent component tends to well approximate the original time series, making it possible to interpret $v_{1}$ as predictive of the measured time series.

## Results

HAVOK analysis was applied to the preprocessed time series through the havok() R package (Moulder et al., 2021). 7,021 HAVOK models were fit in the *stackmax* (number of shift-stacked rows in the Hankel matrix) and *r* (model degree/truncation parameter) hyperparameter space where *stackmax* ranged from 3 to 120, and *r* included all the possible model degrees within each *stackmax* (i.e., *r* ranged from 2 to *stackmax* within each *stackmax*). Derivatives of the truncated V-transpose matrix were estimated with fourth order central difference (FOCD).

Models with squared correlation $R^{2}>.7$ between the first latent time series of the data ($v_{1}$) and the predicted first latent time series ($\hat{v_{1}}$), and models that showed any potential to have a considerable improvement in their fit due to sparsification of the coefficient matrix (see Martynova & Boker, In Process, to identify such models) were sequentially sparsified and refit. Each step of sequential sparsification included setting the smallest magnitude coefficient in the model coefficient matrix to 0 and refitting the regression model while holding that coefficient (and any coefficients set to 0 at previous sparsification steps) at 0. These sparsification steps were repeated until an entire column of the coefficient matrix was set to 0. HAVOK models generated at each step of sequential sparsification were recorded and constituted the third dimension, *λ*, of the hyperparameter space along with *stackmax* and *r*.

### Candidate Model Selection.

Four best-fitting models were selected at four distinct timescales according to the following criteria:

1. Only models that had $R^{2}>.9$ and model degree *r* of 4 or higher were considered, with higher model degrees preferred within a cluster of well-fitting models (Martynova & Boker, In Process). All model components as well as the model structure of the models that fit these criteria were thoroughly examined.
2. In order to minimize the chances of selected models having spuriously good fit, spurious model components and spurious model component relationships that might not be representative of the operation patterns of the true underlying system, only models that belonged to all four types of well-fitting ($R^{2}>.75$) model clusters identified below were considered:
  - Clusters that were spatially compact in the hyperparameter space.
  - Clusters that had very similar forcing terms.
  - Clusters that possessed multiple highly similar or almost identical U-modes.
  - Clusters that were similar in the structure of their coefficient matrices.
3. Out of the models that satisfied the above criteria, models that had the best numeric and visual fit between $v_{1}$ and $\hat{v_{1}}$ time series, as well as the least noisy forcing terms and the most distinctly/unambiguously identified forcing events were selected.

Four models were then selected from the models that met the above criteria. Each of the models was selected to be representative of a distinct timescale (have considerably different *stackmax* from the other selected models), to possess a forcing term that was distinctly different from the forcing terms of the other 3 models, and to belong to the dominant forcing term cluster in the corresponding timescale range.

### Final Model Selection.

One of the 4 selected models was selected for extended analysis and forcing event alignment. This model was chosen for the following reasons.^2^

1. It was a member of the largest continuous cluster of well-fitting models ($R^{2}$ between $v_{1}$ and $\hat{v_{1}}$ exceeding .75) in the hyperparameter space, thereby maximizing the chance of it being representative of the true underlying system’s operation patterns.
2. It had the forcing term with the most distinct and sharp peaks and troughs whose maxima indicated the most precise timing of forcing events.
3. The average frequency (the timescale) of the extracted forcing term events was the most appropriate for alignment with relevant political events.
4. The forcing term had identifiable directionality that aligned with the context, i.e., the peaks identified forcing events that increased Twitter troll activity on selected topics, while troughs identified forcing events that potentially decreased it.

**Figure 4.**
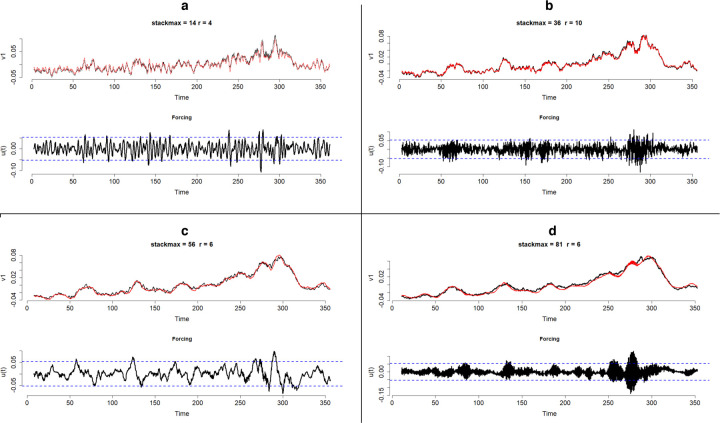
Output of four selected well-fitting HAVOK models at four different time scales. a) stackmax=14 (3.5 days), r=4. b) stackmax=36 (9 days), r=10. c) stackmax=56 (14 days), r=6. a) stackmax=81 (20.25 days), r=6.

Application of HAVOK analysis to the preprocessed time series resulted in a number of well-fitting ($R^{2}>.75$ between $v_{1}$ and $\hat{v_{1}}$) and extremely well-fitting ($R^{2}>.9$ between $v_{1}$ and $\hat{v_{1}}$) models that were available at different time-scales. Figure 4 plots four selected HAVOK model results that focused on four distinctly different time scales. The bottom plot in each of these four HAVOK models depicts forcing terms with a 2 SD threshold plotted here for reference. The black line in the top plots represents major latent component of the measured time series extracted by singular value decomposition, while the red line represents major latent component of the time series predicted by the HAVOK model. Major latent time series resemble a smoothed version of the original measured time series, with the degree of smoothing depending on the chosen number of rows in the Hankel matrix, which is one of the hyperparameters in HAVOK, called *stackmax*. As you can see, the higher is the *stackmax* and, correspondingly, the number of days in the kernel, the more smoothed the time series are, and in turn the slower is the time scale of events that the model focused on. For instance, a *stackmax* of 36, given that we have 4 measurements per day is equivalent to 9 days. So it means that the model with such *stackmax* identifies systematic patterns in shifting 9-day long kernels. The longer the kernel, the more prominent and lower frequency events the HAVOK model will focus on, with longer kernels resulting in more smoothing and more focus on slower time scales.

As can be seen in Figure 4, these four HAVOK models reproduce the major latent time series component of the measured time series very accurately, with the squared correlation between the first latent component of the time series ($v_{1}$) and model predicted first latent component ($\hat{v_{1}}$) in all shown cases exceeding .9. The model with stackmax=14, r=4, λ=.002 (Figure 4a) had $R^{2}$ between the first latent time series of the data ($v_{1}$) and the first latent time series from the HAVOK model ($\hat{v_{1}}$) of .92; the model with the stackmax=36, r=10, λ=.1242 (Figure 4b) had $R^{2}=.99$, the model with the stackmax=56, r=6, λ=.006 (Figure 4c) had $R^{2}=.98$, and the model with stackmax=81, r=6, λ=.04 (Figure 4d) had $R^{2}=.98$.

The $R^{2}$ between the extracted and predicted first latent component and the original time series were low in all models and decreasing with an increase in *stackmax* (lowering frequency of the time-scale). The model with stackmax=14, r=4, λ=.002 (Figure 4a) had $R^{2}$ between the original time series and $v_{1}$ of .26, and $R^{2}$ between the original time series and $\hat{v_{1}}$ of .34. The model with stackmax=36, r=10, λ=.1242 (Figure 4b) had $R^{2}$ between the original time series and $v_{1}$ of .22, and $R^{2}$ between the original time series and $\hat{v_{1}}$ of .23. The model with stackmax=56, r=6, λ=.006 model (Figure 4c) had $R^{2}$ between the original time series and $v_{1}$ of .20, and $R^{2}$ between the original time series and $\hat{v_{1}}$ of .22. The model with stackmax=81, r=6, λ=.04 model (Figure 4d) had $R^{2}$ between the original time series and $v_{1}$ of .20, and $R^{2}$ between the original time series and $\hat{v_{1}}$ of .21.

These estimates are low due to the high frequency intraday fluctuations being smoothed over and discounted in the major latent component of the time series. As the *stackmax* and, accordingly, the length of the timescale and the level of smoothing increase, the $R^{2}$ estimates between the extracted and precticted major latent time series and the original time series decrease. This indicates that the high frequency intraday fluctuations in the tweet time series will not be predicted well by the models that focused on slower timescales. Instead, however, each model will be appropriate for predicting events at its corresponding timescale, which is reflected by the >.9 coefficients between $v_{1}$ and $\hat{v_{1}}$.

Many well-fitting models were available at different time-scales. At some timescales no well-fitting models were available, potentially showing that those particular timescales were not aligned with the timescale of events that forced the system. In the context of the troll data, those would be the time-scales of events that did not incite troll tweets. On the other hand some timescale ranges had multiple well-fitting models available, implying that those time-scale ranges align with the frequencies of the most prominent events that affected the system.

Out of these 4 models, the one with the kernel of length 16 days was surrounded by the densest cluster of well-fitting models in the hyperparameter space implying that this model is potentially appropriately capturing the time-scale of the most influential events that incited active troll tweets.

**Figure 5.**
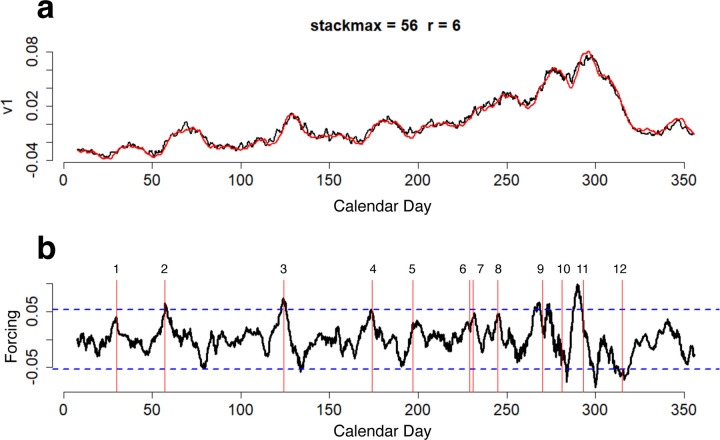
Plot of results of the final selected model. (a) SVD derived major latent time series of volume of right wing troll posts from IRA in black and HAVOK-predicted latent time series in red. (b) Nonlinear forcing term in black, blue dotted 2 standard deviation lines, and 12 influential events: 1) Iowa Caucuses, 2) David Duke (KKK) and Chris Christie endorse Trump, 3) Ted Cruz drops out and Trump is declared the presumptive Republican nominee, 4) Trump’s personal attacks on Hillary Clinton, 5) Trump officially introduces Mike Pence as his running mate, 6) Trump’s speech on fighting terrorism, 7) Trump shakes up campaign with new manager and CEO, 8) Trump’s immigration speech, 9) 1st Presidential Debate, 10) 2nd Presidential Debate, 11) 3rd Presidential Debate, 12) Preliminary election results announced.

Figure 5 is a close up view of the model results. Squared correlation was .98 and the forcing term had very sharp and prominent peaks and throughs. But do these high amplitude peaks align with the political events that had the power to force the right-wing trolls to actively promote Trump on Twitter? Figure 5-b identifies and labels 12 major events during calendar year 2016 that had substantial political relevance.

In this particular system the peaks correspond to the events that forced active Trump promotion on Twitter (e.g., Duke’s and Christie’s endorsement), while troughs correspond to sharply decreased Trump promotion activity (e.g., after the announcement of the preliminary election results). This interpretation is relevant only to this particular system, and will not necessarily be the case in other systems or even models of the same system. The meaning of the forcing term directions depends on the shape of the U-mode of the forcing term, its relationship to the other U-modes, as well the structure of the system’s attractor.

It is noteworthy that the forcing peaks and troughs exhibit a different alignment pattern during the presidential debates. Forcing is active immediately before and right after each debate, but not during the debates, as might have been expected from the alignment of other events.

**Figure 6.**
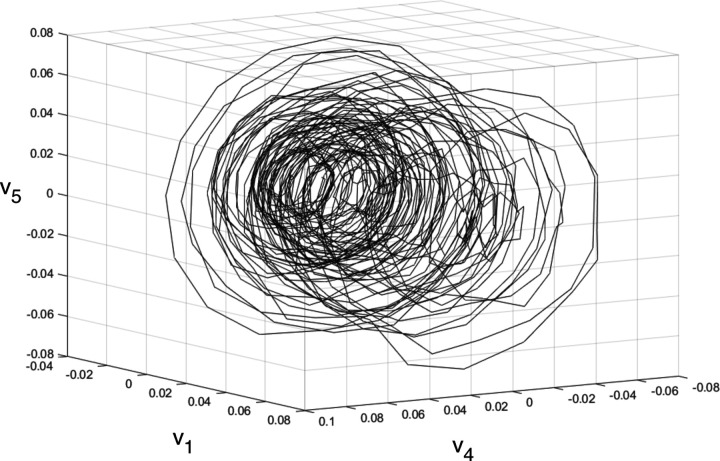
Hectadecagonal (16-sided) attractor reconstructed from embedded dimensions $v_{1}$, $v_{4}$ and $v_{5}$.

Model results can be viewed in higher dimensions in a form of the system attractors pictured in Figure 3. Figures 3-a through 3-c represent the attractor of the modeled system along the first 3 embedded dimensions. It is a spiral attractor with prominent quadrangular cycles and a narrowing in the middle of the spiral cone. Highlighted red regions represent regions with high nonlinear forcing activity (where forcing values exceed +− 1 SD of all extracted forcing term values). Certain regions of the attractor contain higher concentrations of nonlinear forcing as highlighted in Figure 3-c. These regions might be interpreted as attractor segments in state-space where nonlinearities have a higher chance of occurring. Figure 6 plots the system attractor along the 1^*st*^, 4^*th*^ and 5^*th*^ embedded dimensions ($v_{1}$ vs $v_{4}$ vs $v_{5}$). It depicts a spiral attractor with prominent 16-sided cycles. The nature of these hectadecagonal and quadrangular cycles discernible in the attractor can be more clearly understood by examining the U-modes of the model.

**Figure 7.**
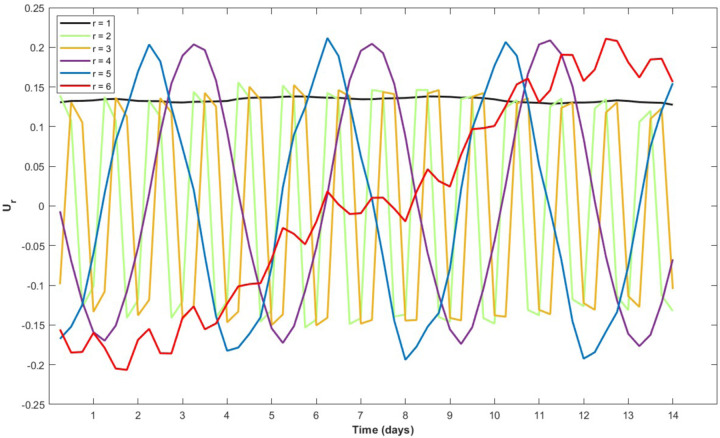
U-modes of the final selected model. U-modes 2, 3, 4, and 5 are sinusoidal. U-modes 2 and 3 align with daily cycles and U-modes 4 and 5 align with 4 day cycles.

The U-modes of the selected model are presented in Figure 7. The 1^*st*^ U-mode is approximately linear with mild curvatures. Such a structure of the first mode is typical for HAVOK models, representing a convolution kernel that extracts an equivalent of the filtered and smoothed ‘position’ (i.e., 0th derivative) of the original time series. Atypically for HAVOK models, however, none of the following sequential derivatives representing U-modes are present. Instead, U-modes 2 through 5 possess phase-shifted sinusoidal shapes that resemble Fourier modes. U-modes 2 and 3 possess sinusoidal shapes of the same frequency lagged by π/2. U-modes 4 and 5 are also represented by a pair of equifrequent sinusoids lagged by π/2. The cycles that they represent are evident in the attractor plots with the U-modes 2 and 3 generating the quadrangular cycles in Figure 3, and U-modes 4 and 5 generating the hexadecagonal cycles in Figure 6. The quadrangular cycles, and, correspondingly, U-modes 2 and 3, represent daily cycles, as the four vertices in each cycle represent the 4 measurements that were collected per day. Analogously, the hexadecagonal cycles, and, correspondingly, U-modes 4 and 5, represent 4-day cycles, as sixteen vertices, when divided by 4 measurements per day result in 4 days. The structure of these first five U-modes that represent linear components in the selected model are not unique to the selected model. These U-modes are present in all 4 models demonstrated in Figure 4, implying that they are timescale universal and potentially represent stable components of the “troll factory” functioning that are invariant relative to external events occurring at different timescales.

The 6^*th*^ U-mode, which represents the kernel of the forcing term, is unique to the selected model (and the neighboring models clustered in the hyperparameter space). Its shape resembles a logistic curve with sinusoidal daily oscillations that oscillate in the opposite direction from the daily sinusoids in U-modes 2 and 3 in the first 1/3^*rd*^ of the kernel, in the same direction as the daily sinusoids in U-modes 2 and 3 in the last 1/3^*rd*^ of the kernel, and phase transition between the two in the middle 1/3^*rd*^ of the kernel (see Figure 7). This kernel shape likely indicates that events forcing the system at this timescale can be characterized by an increase or decrease in the moving average of the original measured time series in combination with a disruptive effect on the typical structure of the daily cycles. That disruption is followed by a phase shift in the daily cycles and eventually their normalization. The phase shift in the daily cycles can be witnessed in Figure 3-c, where the quadrangular cycles in the nonlinear regions (where the forcing is active) are positioned at a different angle than the quadrangular cycles in the linear regions (unaffected by the forcing).

The relationships between the latent time series components $\left(v_{1},...,v_{6}\right)$ that correspond to the six U-modes can be viewed in the state-space equation

(2) $$\frac{d}{dt}\left[\begin{array}{l} v_{1}\\ v_{2}\\ v_{3}\\ v_{4}\\ v_{5} \end{array}\right]=\left[\begin{array}{rrrrr} 0.0000 & -0.0262 & 0.0000 & -0.0119 & 0.0656\\ 0.0297 & 0.0000 & 5.2999 & -0.0230 & 0.0000\\ 0.0000 & -5.2978 & 0.0000 & 0.0456 & -0.2392\\ 0.0000 & 0.0000 & -0.0472 & 0.0000 & -1.5467\\ -0.0649 & 0.0000 & 0.2388 & 1.5466 & 0.0000 \end{array}\right]\left[\begin{array}{c} v_{1}\\ v_{2}\\ v_{3}\\ v_{4}\\ v_{5} \end{array}\right]+\left[\begin{array}{r} 0.0399\\ 0.0000\\ -0.1155\\ 0.0000\\ 0.0000 \end{array}\right]v_{6},$$

where $v_{1}$ through $v_{6}$ represent rows of the V-transpose matrix, and $\frac{d}{dt}$ signifies first derivative with respect to time. The coefficient matrix is sparse with conspicuous structural relationships. The forcing term loads directly onto $v_{1}$, the major latent component of the measured time series, and $v_{3}$, one of the daily cycle components. From $v_{1}$ and $v_{3}$ forcing propagates to the remaining linear components $v_{2}$, $v_{4}$ and $v_{5}$ (the coefficients connecting $\frac{d}{dt}v_{2}$, $\frac{d}{dt}v_{4}$ and $\frac{d}{dt}v_{5}$ to $v_{1}$ and/or $v_{3}$ are non-zero). The highest magnitude coefficients are those that connect $\frac{d}{dt}v_{2}$ and $v_{3}$ (5.2999), and $\frac{d}{dt}v_{3}$ and $v_{2}$ (−5.2978), as well as $\frac{d}{dt}v_{4}$ and $v_{5}$ (−1.5467) and $\frac{d}{dt}v_{5}$ and $v_{4}$ (1.5466). Their high magnitudes represent the high interrelation between the pairs of cyclic components of the same frequency, i.e. the two daily cycle modes, and the two 4 day cycle modes. The next pair of largest magnitude coefficients is connecting $\frac{d}{dt}v_{3}$ and $v_{5}$ (−0.2392), and $\frac{d}{dt}v_{5}$ and $v_{3}$ (0.2388). These coefficients connect daily and 4-day cycles through their two components (one from each pair) that oscillate in phase (see U-modes 3 and 5 in Figure 7).

## Discussion

Many well-fitting HAVOK models were found, representing different timescales. These models were clustered across and within different timescales in the stackmax×r×λ hyperparameter space. Moreover, well-fitting models tended to possess overlapping U-mode structures, model structures and forcing terms. The cluster dominance of the elements of these model features is likely to be proportional to their importance to the true underlying system. For instance, timescale (*stackmax*) regions that contain the largest and densest well-fitting model clusters are likely the most representative of the internal troll factory functioning and/or the timescales of external events that incited troll posting activity. Analogously, timescale regions that contain few well-fitting models are likely to be indicative of timescales that had no particular importance for the troll operation. When the models in low density timescale regions contained little similarity to the nearest dominant clusters, they were likely to indicate the transitions between dominant model structures at distinct timescales.

Figure 4 depicts the results of four dominant models that represent four distinct timescales, with **Model a** focused on the timescale equivalent to 3.5 days (stackmax=14), **Model b** to 9 days (stackmax=36), **Model c** to 16 days (stackmax=56), and **Model d** to 20.25 days (stackmax=81). These four models have distinctly different forcing terms, which implies events forced the system at different timescales. Although some of the differences between the forcing terms could be generated by noise in the forcing term, this seems unlikely since all four models had model degree r≪stackmax and their coefficient matrices were sparsified. The reason for this conclusion is that the lower the model degree *r* relative to *stackmax*, the more elements of the SVD matrices are truncated, thus eliminating more potentially noisy components from the final HAVOK model. Sparsification of the model coefficient matrix serves as yet another noise filtering step in cases where the truncation was insufficient for eliminating all the noise (Martynova & Boker, In Process). Given that the four models in Figure 4 had 71–93% of their SVD matrices truncated, and 42–87% of their model coefficient matrices sparsified out, the remaining forcing terms are unlikely to be contaminated by a substantial amount of noise. Hence, we believe that the forcing terms presented in the plots predominantly contain information about real world events that forced the analyzed troll operation system.

The alignment of the extracted forcing events in **Model c** with the major politically relevant events during the presidential campaign is evident in Figure 5. This alignment of the forcing time series is a striking feature of the HAVOK analysis. The detected event-synchronized features are not apparent by eye in the original time series shown in Figure 2. They are also unapparent in smoothed versions of the original time series. It is important to emphasize that even though some of the identified forcing events are in approximate alignment with the local maxima/minima of the smoothed time series (which can be witnessed in Figure 4, where $v_{1}$ is analogous to the smoothed and filtered original time series), the occurrence of these local maxima/minima in the measured time series is not what the forcing identifies. Forcing identifies the events that are likely to have induced changes such as the emergence of local maxima/minima. Note that in Figure 5, forcing peaks tend to precede the local maxima in $v_{1}$. These forcing peaks identify the timing of events that incited increases in troll activity, followed by troll response increases in $v_{1}$. If the political events were simply aligned with the peaks in the original or smoothed measured time series, they would have been identified incorrectly, or not identified at all as there would have been no significant events at these times. Direct alignment with time series’ maxima/minima seems to be a common practice in event identification in the social sciences. Hence, we find it important to emphasize HAVOK’s ability to identify the timing of events that *precipitate* changes in the measured time series. When used correctly, this feature of the HAVOK analysis can aid in detection of events that influence the measured system.

In addition, in the context of the analyzed troll activity model, this feature of the HAVOK analysis can inform us about troll reaction speed (i.e., time from the onset of the forcing event to the escalation of the troll posting activity), and the manner in which the trolls tended to initiate and terminate their posting activity. Increased posting activity sometimes dissipated gradually as it lost relevance whereas sometimes the troll posting volume declined due to a second forcing event. In Figure 5, the initiation and termination of the troll activity bursts around the 2^*nd*^ and 3^*rd*^ presidential debates were likely caused by the evident forcing events. It may be that premeditated strategies by the troll governance determined optimal times for topical shifts between the debates.

Insight into features of the troll operation can be gained from the dominant patterns of U-modes and model structures. U-mode structures that pervade clusters of well-fitting models are likely to have a meaningful structure that is representative of either the internal functioning of the troll factories, or the influence of external events on the troll activity patterns. For instance, U-modes can capture cyclicity and patterns of the rate of change in the troll activity. Repeating elements of the model structures that define the relationships between the U-modes can further inform us on the nature of connections between the different types of cyclicities and rates of change, as well as their stability and relationship to the external forcing. Distribution of the dominant U-modes across different timescales can also distinguish those that are timescale independent from those that are timescale specific.

### U-modes and the Russian work week

The U-modes of **Model c** suggest that day-night and four-day cycles were the most important dynamics, suggesting coupling with daily and weekly cycles rather than intrinsic dynamics of the troll postings. That is to say, there was less evidence that one posting led to the next in a deterministic way (intrinsic dynamics) than there was evidence that the postings were coupled to day-night cycles and four-day cycles. Day-night cycles and four-day cycles were present among the U-modes of all four models at four different timescales pictured in Figure 4. In **Model c**, U-modes 2 & 3 are aligned with day-night cycles and modes 4 & 5 are aligned with four-day cycles. As can be seen in Figure 7 the two cycles are captured by pairs of sinusoidal shapes lagged by π/2, providing evidence that Fourier modes can be detected by HAVOK analysis in addition to sequential derivatives / Legendre polynomials (Hirsh, Ichinaga, Brunton, Nathan Kutz, & Brunton, 2021). Due to the pervasive presence of these cycles across dominant models at all timescales we hypothesize that these cycles have a substantive meaning.

We explored this hypothesis by locating information from troll factory insider interviews. There is an 8 to 12 hour difference in time zones between the Moscow area in Russia and the USA. To be able to realistically align their tweet patterns with waking time in the USA, Russian trolls would need to work at night, which was confirmed by insiders (Volchek, 2021b, 2021a). These reports claim that there were 12 hour work shifts: a night shift and a day shift. These insiders also reported that they had a 2-by-2 work week, which is the most common work structure in Russia when night shifts and no-weekend jobs are involved. In a 2-by-2 work week, people work for 2 days then are off for 2 days. Thus one team of people worked on days 1–2, 5–6, etc. and a different team worked on days 3–4, 7–8, etc. Thus there would be 4 active teams: Team A would work days for the first 2 days and team B would work nights for the first 2 days. Then team C would work days for the second 2 days and team D would work nights for the second 2 days. We propose that U-modes 2 & 3 capture the 12-hour shifts: peaks and troughs in the sinusoidal 2 & 3 U-modes capture different troll-worker teams at work. Similarly, we propose that U-modes 4 & 5 capture the change in worker teams every 2 days.

The forcing term is out of sync (fluctuating in the opposite direction) with the 3rd U-mode daily cycles for the first one third (visible in the U-mode plot and r=−0.42), in sync (containing daily cycles in the same direction) for the last one third (visible in the U-mode plot and r=0.43), and phase transitioning in the middle one third (r=0.01). The model coefficient of the forcing term that loads on the 3rd U-mode daily cycles is relatively large and negative. This likely implies that occurrence/onset of prominent political events that force the system is accompanied by a disruptive effect on the regular structure of the daily (within 12-hour work shift) tweet posting cycles. One explanation for this disruptive effect is if the beginning of a normal 12-hour work shift cycle involves little posting. According to the insider interviews, trolls receive folders with information on current events and topics of concern at the beginning of a shift and they take time to read and prepare before starting to actively post. At shift end, these reports say that the workers are tired and post less. However, the occurrence of a forcing event might induce the workers to hurry and post about that event while it is still relevant even if this is not their typical peak productivity hours, causing *irregularities* in the cycles representing 12-hour shifts. The directional flip in the forcing kernel shape in the second half might indicate the normalization of the regular daily work schedule after a panic posting episode.

The forcing term has no direct loadings onto the four-day cycles in the model which implies that political events do not directly affect the 2-by-2 work week structure. However, the model structure indicates that changes in the 12-hour shift cycles in turn generate changes in the 2-by-2 work week cycles (loadings from $v_{3}$ onto $v_{4}$ and $v_{5}$ are not zero, see Equation 2). According to the insider interview, trolls had the opportunity to request change in their shift from night to day or vice versa (Volchek, 2021a). Given this knowledge, the indirect effect might indicate that the irregularities caused by the forcing events in the 12-hour shift cycles affected troll workers in ways that increased their chances of requesting a shift change, in turn changing their work group. Such a work group change also aligns with the phase shifting property of the forcing term discussed above. We hypothesize that prominent political events might provide more clarity on potential posting content, as a result making it easier for the troll workers to meet their reported daily quota of 120 posts. Some trolls might see prominent political events as an opportunity and work beyond their daily quota in return for bonus payments. Others might choose to leave work early after the expedited quota completion. The first scenario might have caused trolls to overwork, encouraging them to request a shift change in the direction that would allow for a longer rest period. The second scenario might have had an opposite effect, encouraging the trolls to request a shift change in the direction that would decrease the time between the expedited and the following shift. In either scenario, anomalies caused by the forcing events in the 12-hour cycles would encourage trolls to change their work group captured by the segments of the 4 day cycles, thereby altering the 2-by-2 work week structure.

## Conclusions

This article serves as evidence that HAVOK analysis, when combined with topic modeling using DynEGA, can reverse engineer Russian manipulation strategies from text. Topics used to influence the online debates, forcing events that aligned with politically relevant events and incited changes in troll activity, and troll work cycles (day-night, and 2-by-2 work week) were all detected and interactions between them were explored.

When applied across a range of hyperparameters, HAVOK has been shown to reveal the multi-timescale nature of the troll tweeting system with evidence that events forced the system at different timescales. HAVOK was able to determine many well-fitting models across all timescales of interest. It exhibited model fit patterns that distinguished the timescales that were representative of the troll factory functioning from the timescales that held no particular importance for the troll operation. The distribution of model features in the hyperparameter space allowed identification of features of the system that were timescale-universal from those that were timescale-specific. HAVOK’s separation of linear and nonlinear model features also allowed observation of the evolution of the interactions between timescale-specific external nonlinear forcing events and internal linear troll functioning patterns across timescales, making it possible to identify differences between trolls’ reactions to events at different time-scales.

HAVOK identified the timing of nonlinear events that forced troll activity at different timescales. These nonlinear forcing events aligned with real world politically relevant events with striking precision. The forcing events precipitated changes in the measured time series of topical troll tweeting activity. The precipitation feature of the forcing events allowed determination of troll reaction speed patterns (i.e., time from the onset of the forcing event to the escalation of the troll posting activity), and the manner in which the trolls tended to initiate and terminate their posting activity. The dominant model provided directional meaning for the forcing eventsŮforcing peaks preceded surges in troll tweeting activity, and forcing troughs preceded ebbs in the troll tweeting activity. The combined knowledge of the timing, the real world event alignment, and the precipitation and directionality of the forcing events permitted realistic explanations of the mechanisms underlying governance of the troll factory operation.

HAVOK analysis provides a mathematically elegant state-space model of this system’s functioning. The model contained meaningfully interpretable structural components that could be mapped onto corresponding components of the real world troll operation system. The U-mode components, which typically extract the patterns of the rates of change in a form of sequential derivatives as Legendre polynomials in HAVOK models governed by intrinsic dynamics, instead captured pairs of phase-shifted cyclic components analogous to Fourier modes. This is evidence that HAVOK possesses an inherent ability to detect cyclic bases in a form of Fourier modes, when the analyzed system is coupled to stationary external forcing. The domination of the cyclic components across Russian Twitter troll activity models at all timescales suggests that coupling with the detected cycles rather than intrinsic dynamics of the troll postings were the most important dynamics of the analyzed system. The alignment of the detected cycles with the Russian troll factory operation cycles was confirmed from insider interviews.

Relationships between the cycles, the rates of change, and other basis elements were contained within the HAVOK models as coefficients connecting the latent time series $v_{1},...,v_{r}$ that respectively correspond to the U-mode bases. Model coefficients also indicated how the external forcing term influenced each linear component of the system, and how its influence propagated throughout the linear components over time. HAVOK allowed visualization of the dynamical process of the troll operation system captured in the models in high dimensional attractors in state space. Elements of the system, such as cycles, consequences of their interactions with the external forcing, such as phase shifts of the cycles, as well as distribution of the nonlinear regions in state space can all be observed and analyzed from a visual perspective. When compared across different timescales, the stability of the system’s elements and their relationships captured in the models and attractors could be determined, identifying those that were stable across timescales from those that were timescale-specific.

This insight was achieved by reapplication of the HAVOK analysis across the hyperparameter space. It was surprising to us how reapplication of such a mathematically simple model provided a way to reverse engineer Russian manipulation strategies with impressive detail and alignment with the real world events. These results allow improved theoretical speculation about the future Russian troll activity and a method to quantitatively predict how future forcing events will manifest in the troll behavior. HAVOK models allow one to simulate a system’s response to hypothetical forcing events. Given that forcing events tend to precede events in the response time series, simulated responses generated by data from events in real time could allow for prediction and/or intervention. Simultaneous short-term forecasting of HAVOK components is also possible by the use of HAVOK forecasting extensions (Martynova, Moulder, & Boker, 2022). All of the above can be executed and analyzed at multiple timescales that possess descriptive models representative of a target system, thereby creating a more complete view of the system and increasing chances of correct predictions.

Equally impressive results can be achieved by applying HAVOK across hyperparameter space to any intensive time series that meet the requirements listed in Martynova and Boker (In Process). Although it would be challenging to achieve such results by the direct application of the havok() function due to high computational demands, our recently developed phavok() function surmounts these hindrances allowing for automatic execution of the HAVOK analysis across large areas of the hyperparameter space with substantially reduced computational time. phavok() is a new function in the havok R package (Moulder & Martynova, 2020) that comprises a parallelized and optimized version of the HAVOK procedure with automated hyperparameter search. The phavok() function is much faster than havok() and can run multiple models simultaneously across different desired or randomized sets of hyperparameters and generate model fit surfaces across sets of hyperparameters in a feasible amount of time. It allows fitting millions of models in a feasible amount of time and thereby improving the chance of finding a well-fitting model. If one possesses a time series with sufficient observations and of reasonable reliability, we encourage downloading and trying the havok package. It is likely that the results will be interesting and surprising.

## Supplementary Material

Supplement 1

## Footnotes

1 For a more extensive tutorial than we can supply here, please refer to Moulder, Martynova, and Boker (2021).

2 Candidate and final model selection criteria served as a guidance in the model selection process rather than strict and immalleable rules.
