# Supplementary material for: Playing HAVOK on the Chaos Caused by Internet Trolls
Source: Res Sq. 2023 Apr 25:rs.3.rs-2843058. Preprint. [Version 1] doi: 10.21203/rs.3.rs-2843058/v1 (PMC10168470; doi:10.21203/rs.3.rs-2843058/v1)
Supplement: Supplement 1 [file NIHPPrs2843058v1-supplement-1.pdf]

Supplementary Files

This is a list of supplementary files associated with this preprint. Click to download.

- TrollsCodeSupplement.r
- results.all.trolls.rdata
